# Subcritical Determination of the Frenkel Line in Liquid Nitrogen, the Emergent Final Picture, and a Universal Equation for the Coordination Number of Real Fluids

**DOI:** 10.1021/acs.jpcb.5c00018

**Published:** 2025-03-25

**Authors:** Ciprian G. Pruteanu, Ayobami D. Daramola, Marcin Kirsz, Cerian E. A. Robertson, Luke J. Jones, Tianrui Wang, John S. Loveday, Graeme J. Ackland, Oliver L. G. Alderman, John E. Proctor

**Affiliations:** † SUPA, School of Physics and Astronomy and Centre for Science at Extreme Conditions, The University of Edinburgh, Edinburgh EH9 3FD, United Kingdom; ‡ Materials & Physics Research Group, SEE Building, University of Salford, Manchester M5 4QJ, United Kingdom; § Photon Science Institute & Department of Electrical & Electronic Engineering, University of Manchester, Manchester M13 9PL, United Kingdom; ∥ ISIS Neutron and Muon Source, Rutherford Appleton Laboratory, Harwell Campus, Didcot OX11 0QX, United Kingdom

## Abstract

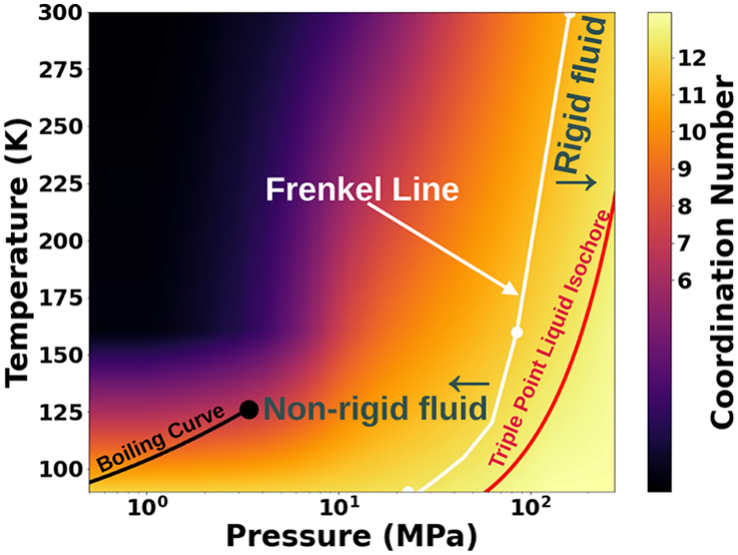

We performed a series of neutron scattering experiments on deeply subcritical liquid nitrogen at 90 K (0.7*T*_*C*_). Our findings, when taken together with our previous results at 160 K (1.27*T*_*C*_) and 300 K (2.4*T*_*C*_), allow the Frenkel line phenomenon to be characterized in a reliable and consistent manner over an extremely broad temperature range, extending into the subcritical regime. Through an analysis of local order, we show how the fluid structure changes as the Frenkel line is crossed and present a new method for identifying the line. Our determination of coordination numbers shows a remarkable data collapse when plotted against density. This allows us to produce a universal relationship relating the coordination number to the density of a simple fluid, dictated by molecular/atomic size and its density on the melt line.

## Introduction

The Frenkel line (proposed in 2012)^1^ divides the supercritical fluid state into regions with different behavior. It is sometimes stated that it is where the sample displays liquid-like versus gas-like behavior. One controversial^2^ aspect of this definition is that the line does not intersect with the critical point. Instead, the line was proposed to pass through the critical isotherm *T*_*C*_ at significantly higher pressure than the critical point and, at *T* < *T*_C_, divide the subcritical part of the phase diagram into a rigid liquid and nonrigid liquid region. The Frenkel line was initially proposed to terminate by intersecting with the vapor pressure curve at ca. 0.7–0.8 *T*_C_, then later proposed to continue all the way to the triple point.^3,4^ Experimental observations of the Frenkel line were not made until some time after its proposal and initially focused on the supercritical region.^5−9^ As far as the critical and subcritical regions are concerned, studies remain sparse. Using optical spectroscopy, the Frenkel line was observed at ca. 0.98 *T*_*C*_ and 50 *P*_*C*_ in ethane^10^ and using neutron diffraction, it was observed at a pressure in supercritical nitrogen (1.27 *T*_*C*_)^8^ that appeared to put it on a trajectory to continue into the subcritical region rather than intersect with the critical point.

From atomic-level considerations supported by molecular dynamics has come the idea that the Frenkel line arises from a crossover in the nature of diffusion, from caging—wherein uncorrelated single-molecule motion dominates and the mean free path is similar to the molecular size—to a state with collective motion and longer velocity autocorrelation.^1,11^ This idea leads naturally to highlight the importance of coordination number: a caged atom should have a full coordination shell. In this case, atoms can vibrate around fixed positions, analogous to the in a solid. This is a manifestation of the fact that the structural and dynamical properties of matter are interdependent.

Coordination number is conceptually easy to understand but difficult to define rigorously.^12−14^ Even in situations where a complete model of atomic positions is available, one still has to define some radius within which the “coordinated″ neighbors lie. Typically, this comes from the first minimum in the radial or cumulative distribution function.^15,16^

Along a supercritical isotherm, one expects the coordination number to be proportional to density in the low-density limit, flattening off to “full″ coordination of around 12–14 at high density. The crossover between those limiting behaviours has been proposed and used experimentally as a marker of the Frenkel line,^3,6−8,17^ and these studies have been recently reviewed in ref. (17).

In the present study, we have performed the first neutron scattering measurements attempting to locate the Frenkel line in a subcritical fluid (nitrogen). We find conclusive evidence that the line is still detectable at a temperature of 90 K in N_2_ (0.71 *T*_*C*_ at a pressure about an order of magnitude higher than the boiling pressure), suggesting it does not end at 0.7 *T*_*C*_ as expected from previous considerations. Moreover, the evidence from the present study strengthens the proposal that the line does not end/originate at the triple point. Combining the current measurements with our previous ones at 160 K^8^ and 300 K^6^, we are able to formulate an analytic expression describing the evolution of the coordination number of real simple fluids across their regions of existence, both subcritical and supercritical.

## Methods

### Experimental Procedure

We have performed time-of-flight neutron scattering measurements at the ISIS Pulsed Neutron Source, Rutherford-Appleton Laboratory (RAL), Oxfordshire, UK, using the SANDALS diffractometer, which has a neutron wavelength range of 0.05 to 4.94 Å . Samples of pure N_2_ (Grade N5, 99.999% purity) were loaded into a can made from a null-scattering titanium–zirconium alloy (TiZr) optimized for high-pressure experiments. We used a flat-plate geometry. The sample was held within 6 cylindrical bores of 1.5 mm diameter within the 6.6 mm thick TiZr plate. The size of the neutron beam is 30 mm × 30 mm square. The pressure of the sample was determined by means of a pressure gauge connected to the pressure cell. A closed-cycle refrigerator (CCR) was used to maintain the sample temperature at 90 K for all data points collected. The run times varied between 7 and 9 h, depending on sample density, in order to ensure an equivalent level of statistics and confidence. Background measurements were performed on the empty TiZr can, the empty CCR at 90 K (the temperature of the measurements), a vanadium–niobium (V–Nb) alloy, and the empty instrument at ambient conditions as a background for the V–Nb to enable normalization of the total-scattering pattern. The background correction and normalization procedure was performed using the GudrunN software package.^18^

### Empirical Potential Structure Refinement

The collected data were analyzed using the Empirical Potential Structure Refinement (EPSR) software package.^19,20^ The input density was determined for each pressure point along the 90 K isotherm according to the Span–Wagner equation of state, which is publicly available on the NIST REFPROP database.^21^ EPSR boxes of 1000 N_2_ molecules were created at each density corresponding to the measured pressures. The EPSR default reference potential (RP) for nitrogen is GROMOS (gromos54a7)^22^ atom type “NR”, using an N–N bond length of 1.10570 Å in the isolated nitrogen molecule. The simulation box is first equilibrated using just the reference potential in a Monte Carlo simulation, and then the additional empirical potential (EP) was switched on to improve the fit by driving the Reverse Monte Carlo procedure toward better agreement with the data. Several thousand trials were made, allowing the EP to find the minimum amplitude at which the best fit to the data was achieved. Afterward, the EP was frozen, and the data were sampled for more than 5000 accumulations of the simulation box, while monitoring the convergence of statistical sampling of key quantities of interest. In a similar fashion to our previous studies,^3,6,8,9^ we monitored the pair distribution function *g*(*r*), the coordination number (using the first nonzero minimum in the *g*(*r*) as the cutoff distance for the first neighbor shell), and several other quantities, including the separate intra- and intermolecular contributions to the structure factor.

### Molecular Dynamics Simulations

We also conducted simulations using molecular dynamics (MD) based on the purely theoretical machine-learned interatomic potential,^23^ developed with the open-source Tadah! package.^24^ This potential was trained solely on data from CCSDT(Q) quantum chemistry calculations, yet it demonstrates remarkable transferability, providing an excellent description of all molecular solid phases and the melt curve. The potential is publicly available on the Tadah! website.

The MD simulations were performed using the LAMMPS^25^ simulation package with the Tadah! plugin. The equations of motion were integrated using the velocity Verlet algorithm as implemented in LAMMPS.^26−28^ Calculations utilized the NPT ensemble with a Nosé-Hoover thermostat^29^ and a Parrinello-Rahman barostat.^26^ A time step of 1 fs was maintained throughout. All simulations were first equilibrated for 150 ps, followed by a 2 ns production run. The simulation box contained 2048 molecules. LAMMPS relaxation parameters were set to 0.1 ps for the temperature and 0.5 ps for the pressure. We set the drag to zero for both the thermostat and barostat to maintain energy conservation and avoid spurious contributions to the system’s dynamics. These settings were tested in our previous work and found to show excellent agreement with the experimental phase diagram.^23^

### Criteria for the Identification of the Frenkel Line

As noted in previous diffraction-based work by Prescher et al.^7^ and Pruteanu et al.,^6^ the Frenkel line can be located by the plateauing of the coordination number of the material with increasing pressure. This means of identification also readily discriminates between the Frenkel line and potential Widom lines^8^ when in the vicinity of the critical point. With the aid of a machine-learned classical force field for nitrogen, a mathematical expression^3^ has been formulated that describes this behavior independently of temperature and offers a clear quantitative cutoff for the location of the Frenkel line, namely:
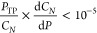
1where P_*TP*_ denotes the triple point pressure, *P* the pressure of the material, and *C*_*N*_ is the coordination number (defined as described above). This criterion has also been used successfully to locate the Frenkel line in Krypton at 310 K^9^ and in square-well potentials representative of colloid–polymer mixtures.^4^

## Results

### The Frenkel Line in Liquid Nitrogen at 90 K

**Figure 1:**
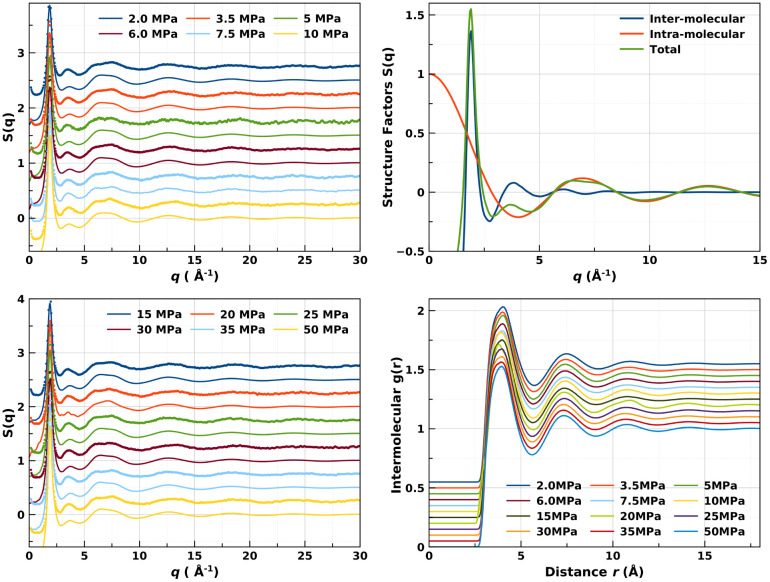
(Left) All raw measured *S*(*q*) patterns (points) and EPSR fits (thin smooth lines) to the *S*(*q*) patterns for nitrogen. Top: lower pressures; Bottom: higher pressures. The fits are depicted immediately below the corresponding raw data set, offset vertically by 0.5 for clarity. (Right) Fitted *S*(*q*) at 25 MPa and its intramolecular and intermolecular contributions (top). Note that the first minimum in the intramolecular overlaps with the second maximum present in the intermolecular partial structure factor. Intermolecular Pair Distribution Functions of nitrogen extracted using EPSR at all pressures investigated in this study (bottom).

The total scattering patterns (*S*(*q*)) collected in the present study are depicted in Figure 1 along with the associated fitted patterns (left panels) and corresponding intermolecular pair distribution functions (bottom-right panel). The structure factors are extremely similar, which is to be expected given that, despite the apparently large pressure range considered (2–50 MPa, a 25-fold increase), the density only varies by ∼12% (751.43 kg/m^3^ and 855 kg/m^3^) between the lowest and highest pressures, respectively).

A complication in obtaining accurate intermolecular pair-distribution functions becomes readily visible upon inspection of Figure 1, top-right panel, which shows the intra- and intermolecular contributions to the measured structure factor. Due to the shape and size of the nitrogen molecule (essentially a “dumbbell”), the intramolecular structure factor has a minimum that almost coincides with the second maximum of the intermolecular structure factor. This situation highlights the importance of carefully accounting for molecular geometry in order to allow the separation of intermolecular structural properties of interest from the properties of the constituent molecules themselves. This is readily available in real-space modeling and structure-solving approaches based on Reverse Monte Carlo techniques (EPSR^20^ Dissolve^30^ RMCPOT),^31^ but it is significantly more challenging in the commonly used procedures to obtain the radial distribution function using a direct Fourier transform from *S*(*q*).

**Figure 2:**
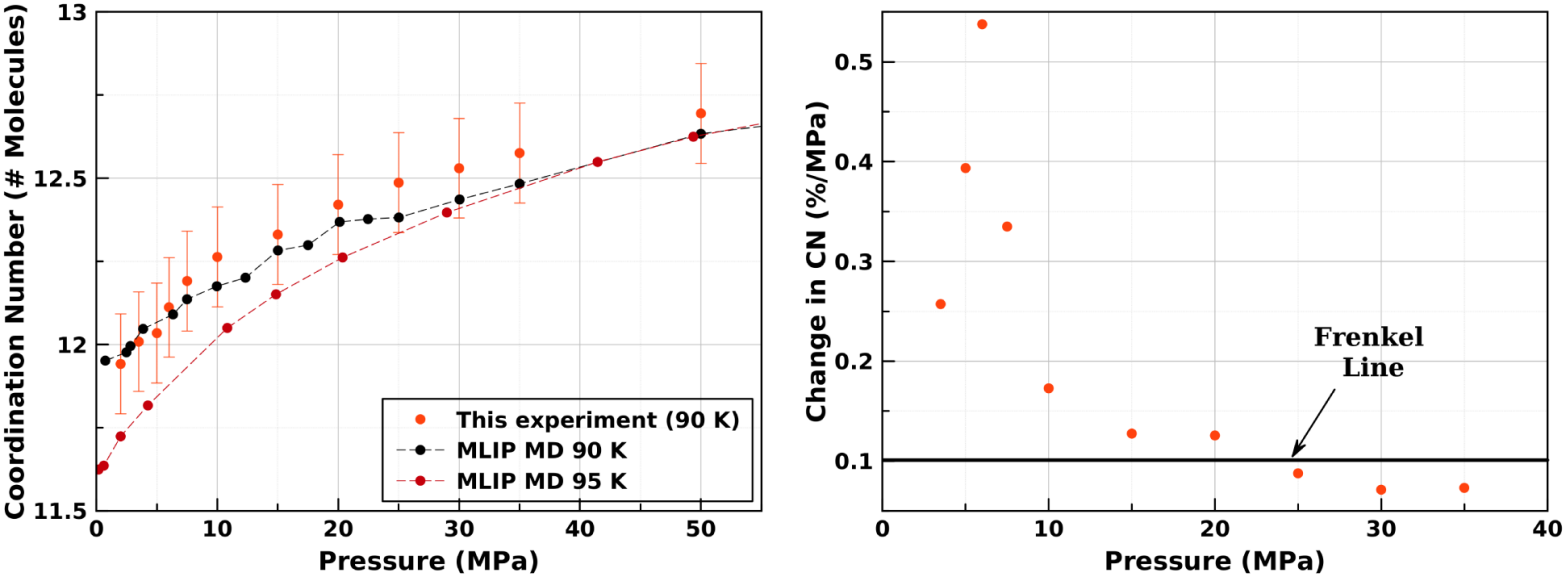
(Left) Coordination numbers of subcritical liquid nitrogen at 90 K, obtained from EPSR radial distribution functions and from molecular dynamics simulations using a machine-learned interatomic potential.^3,23^ A tendency for the *C*_*N*_ to level off above ∼15 MPa is easily visible. (Right) Percentage change in the intermolecular coordination number of N_2_ as a function of pressure.

The application of eq 1(3,4) in the present study indicates crossing the Frenkel line at 90 K under a pressure of 22 MPa (Figure 2), in good agreement with the extrapolation from previous work at 160 and 300 K.

We have also looked at alternative means of identifying the Frenkel line, making use of the full 3-dimensional structures obtained from EPSR. To this end, we have performed Ackland-Jones local environment classifications^32^ on a selection of simulation boxes generated in the course of EPSR sampling. This method “groups” the local environments of the individual atoms/molecules in a material, based on the real-space distribution of their nearest neighbors, into crystalline-like categories (face-centered cubic (FCC), hexagonal close-packed (HCP), body-centered cubic (BCC), etc.). It is complementary to using the pair distribution function because it includes information about three-body configuration and is therefore particularly sensitive to the distribution of local environments present within the material. At the densities considered in the experiment, nitrogen is a freely rotating molecule (and remains so upon freezing at even higher densities,^23^ so the local structure analysis is based on the molecular (center of mass) environments.

**Figure 3:**
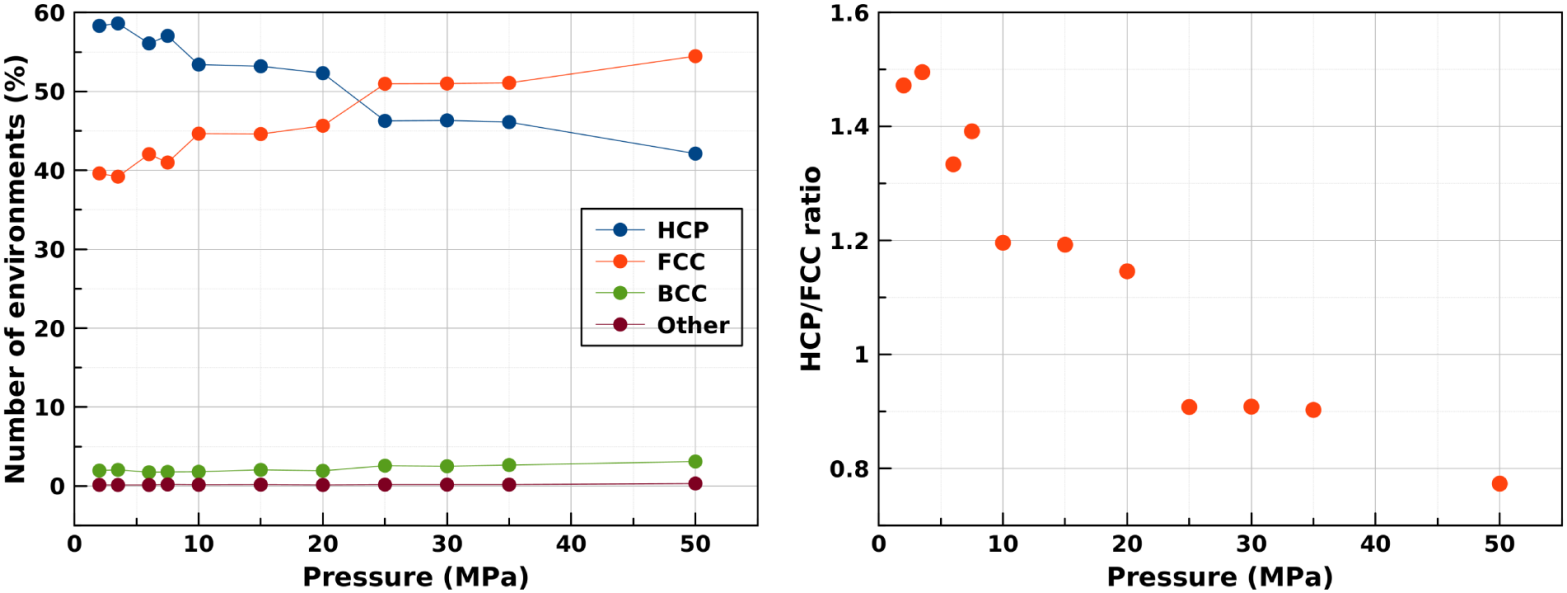
(Left) Fractions of molecules within HCP, FCC, BCC, or other local environments within the 3D structure of the fluid as a function of pressure. (Right) Ratio of atoms in HCP vs FCC local environments (most prevalent environments) as a function of pressure.

There is a clear trend that, with increasing pressure/density, the number of atoms within the fluid identified as being in an FCC-like local environment increases, while the number of those within an HCP-like environment decreases (Figure 3) up to ∼22 MPa. Above this point, the curve flattens noticeably. Significantly, this is the same point identified with the Frenkel line using coordination numbers alone (eq 1)

Owing to the method’s high sensitivity to heterogeneity within the structure, studying this trend, in addition to the trend in coordination number, allows us to obtain a deeper understanding of the changes that take place when the Frenkel line is crossed.

**Figure 4:**
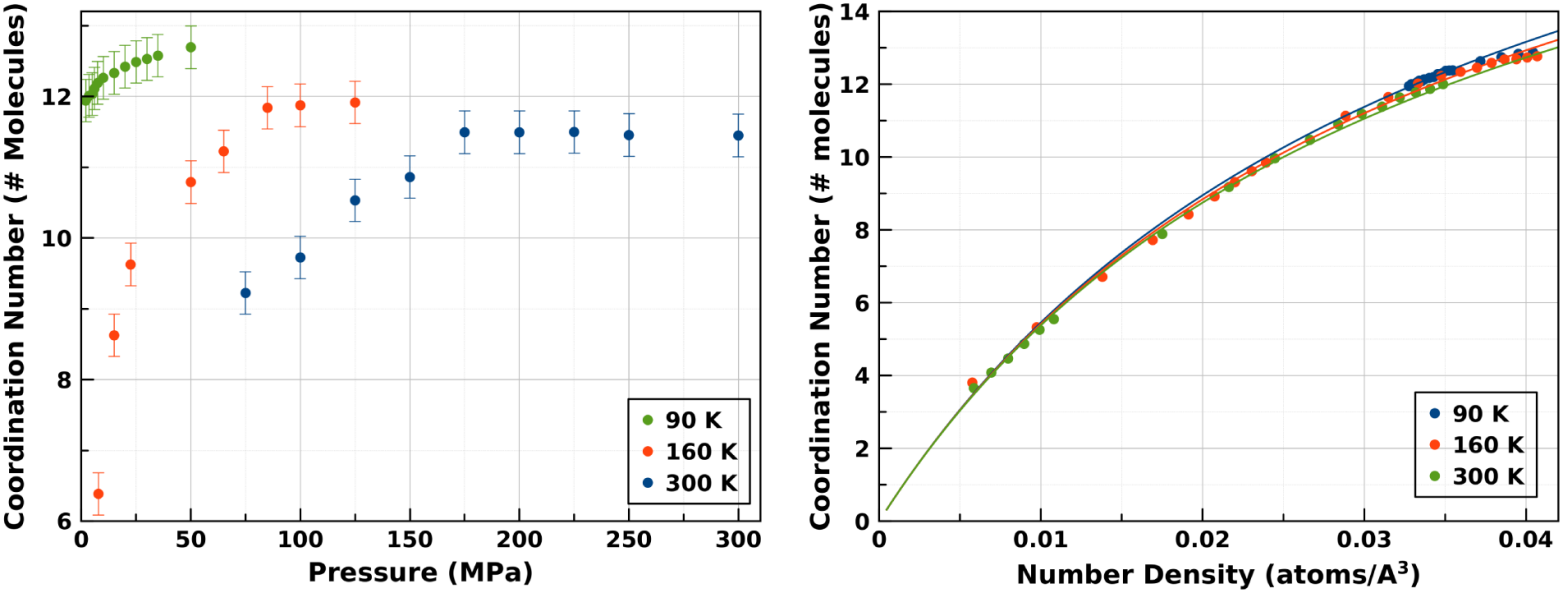
(Left) Behavior of fluid N_2_ across subcritical and supercritical regimes from present and previous neutron scattering measurements. (Right) Evolution of the coordination numbers for supercritical and subcritical N_2_ as a function of density (ρ)—obtained from the MLIP MD simulations.

A complete picture of the Frenkel line throughout the entire phase space available to both the regular liquid and supercritical fluid states emerges when we combine the current data with previous measurements.^3,6^ As readily visible in Figure 4, left panel, the fluid always tends toward a maximally, 12-fold coordinated state with increasing pressure at all temperatures. However, the coordination number and its asymptotic value are systematically lower at higher temperatures.

Our experimental results are in excellent agreement with molecular dynamics simulations carried out using the machine-learned interatomic potential we developed for N_2_ based purely on high-quality quantum chemical calculations involving interactions between two molecules^3,23^ (Figure 4, right).

In order to isolate the effect of pressure from that of temperature on the coordination number of a fluid, it is worth looking at the density–temperature (ρ–*T*) phase diagram rather than the more commonly employed pressure–temperature (*p*–*T*). As the experimental data are rather granular and spaced throughout a large range of pressures/densities due to the presence of the boiling curve at lower pressures, and the impossibility of performing neutron scattering measurements to determine the structure of very low-density fluids, we have used the developed interatomic potential to obtain more fine-grained curves.

Figure 4 (right panel) shows a remarkable data collapse, with the coordination number being a function of density. Close inspection of the figure reveals a small effect due to temperature. The general shape of this evolution invites an attempt to formulate an empirical equation to describe, in analytic form, the dependency of the coordination number of a fluid on its density.

Based on fundamental physical principles, one would expect the coordination number to satisfy two limiting cases: close packing (*C*_*N*_ ≈ 12–14) at high density/pressure and the ideal gas limit (*C*_*N*_ ∝ ρ) at low density/pressure.

This suggests fitting a function of the form:
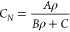


We have seen, to a good approximation, that the liquid *C*_max_ is constant at the melt line. Ensuring this expression actually reaches *C*_max_ at the melting line requires that *C* is weakly density dependent, so one obtains the following:
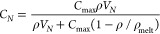
where we introduce the volume around each atom, which we can define to be a “neighbour″, *V*_*N*_. We now have the coordination number, which is equivalent to
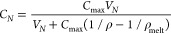
2where *ρ* is the number density.

At low density, *C*_*N*_ → *ρ V*_*N*_ , i.e., the number of atoms in volume *V*_*N*_ is that volume times the density. Also, *C*_*N*_ = *C*_max_ on the melting line, as expected.

*C*_max_ is the liquid coordination number at the freezing point: according to our definition of *C*_*N*_, this is essential. ρ_max_(*T*) is the liquid density at the freezing point, readily available from the equation of state. Finally, *V*_*N*_ could be taken as a fitting parameter and is certainly material dependent, but its definition leads to
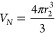
where *r*_2_ is twice the “molecular diameter,” which can be determined as the low-*r* edge of the first peak in *g*(*r*) (2.75 Å). At higher interparticle separations than this, it is always possible to insert another particle in between the two original ones; hence, the latter no longer remain nearest neighbours. Consequently, we will set *r*_2_, and thereby *V*_*N*_, as a temperature- and pressure-independent constant.

This allows the coordination number along each isotherm to be determined without fitting. A weak temperature dependence arises because ρ_max_ is set by the liquid density of the melting line at a given temperature. The comparison of this equation to our data is depicted in the Figure 4 (right panel), and the associated parameters are tabulated in Table 1.

**Table 1:** Parameter Values for the Coordination Number Curves Depicted in Figure 4 for Nitrogen at 90, 160, and 300 K

**Temperature (K)**	*r*_**2**_**(Å)**	**ρ**_melt_**(atoms/Å**^**3**^**)**	**ρ**_melt_ (g/cm^3^)
**90**	5.505	0.0458	1.07
**160**	5.505	0.0479	1.115
**300**	5.505	0.0498	1.16

Figure 4 shows the remarkable data collapse obtained when the coordination number is plotted against the number density for a wide range of pressures and temperatures. Second, we see that eq 2, with parameters that are constrained by their physical meanings, is an excellent fit to the data. A very close inspection of the graph shows a weak temperature dependence. This is accounted for by the model without using temperature-dependent parameters; it arises only from the different densities of the liquid at the melt line, which is fully determined by the equation of state.

**Figure 5:**
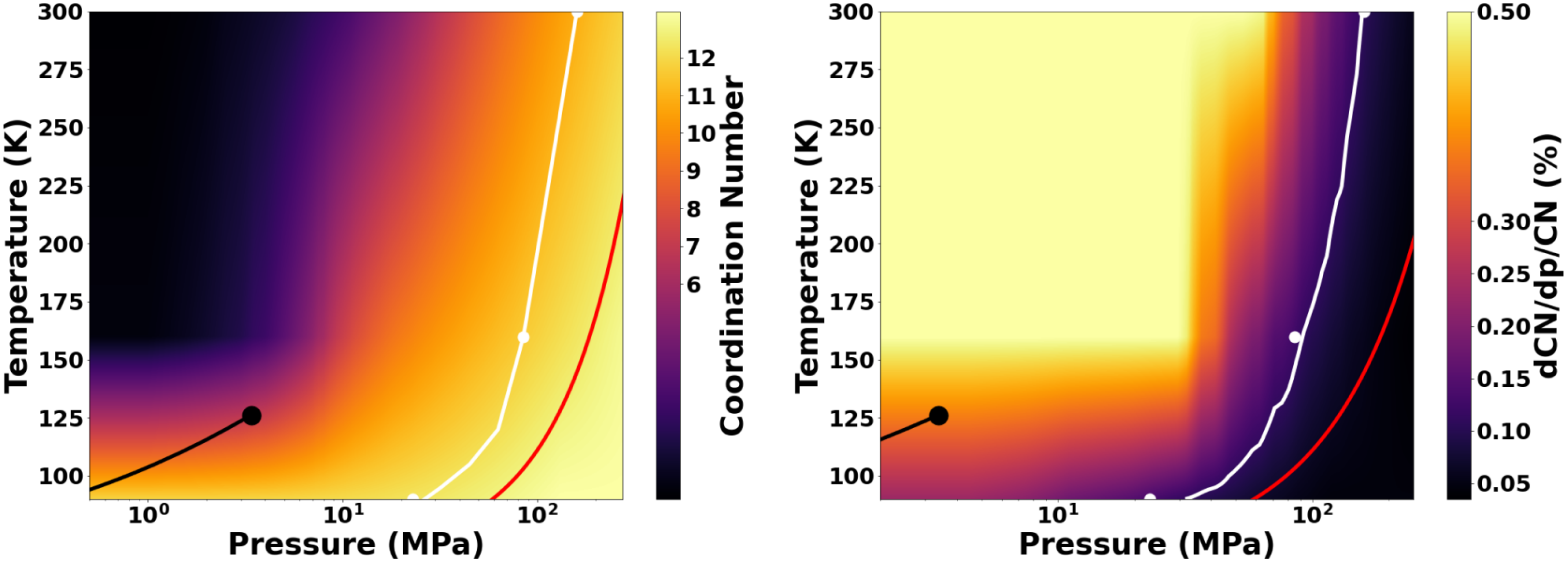
Evolution of the coordination number (left) and its log-derivative with respect to pressure (right) throughout the phase space of subcritical and supercritical N_2_. The log-derivative is used with a maximum cutoff of 0.5 for visual clarity. The white lines represent the Frenkel line as identified using the log-derivative equation (eq 1), and the dots of the same colors denote the points on the Frenkel line as identified from experimental data. The large black dots depict the location of the critical point on the phase diagram and the black lines ending at them the boiling curve. The red lines depict the triple-point liquid isochore. The melting curve is outside the *p*–*T* range of the figures.

Having obtained an analytical formula for the coordination number of a fluid, we can deploy it to obtain a less granular view of the Frenkel line. In Figure 5, we show the evolution of the coordination number and its log-derivative in *p*–*T* space from our experiments to date, along with the stated “crossing” of the Frenkel line and its location throughout the entire phase space calculated from simulations.

## Discussion and Conclusions

The work presented here probes the nitrogen Frenkel line far deeper into the subcritical region than previous works and offers a unique insight when looked at in combination with our previous studies characterizing the supercritical nitrogen Frenkel line. Comparing the observed trends in the coordination number at temperatures of 0.7 *T*_*C*_ (in the present work), 1.27 *T*_*C*_(8) and 2.4 *T*_*C*_(6), it now seems clear that the Frenkel line does indeed extend into the subcritical region toward the vicinity of the triple point.

The extension of the Frenkel line into the subcritical liquid region means that the description of it as a crossover from “gas-like″ fluid to “liquid-like″ fluid must be abandoned. Previous work on the Frenkel line in several supercritical materials has invariably placed it on the high-density, “liquid-like″ side of the Widom linesPC8^8^ but now we have extended the line below the critical temperature: one cannot reasonably claim that the fluid on the liquid side of the boiling curve is “gas-like″.

Our molecular dynamics simulations indicate that a more appropriate microscopic description is one that more closely aligns with Frenkel’s description of a liquid. The fluid changes from a caged structure, where each molecule is surrounded by a full coordination shell, to a more open liquid, where gaps in the coordination allow easy flow.

**Figure 6:**
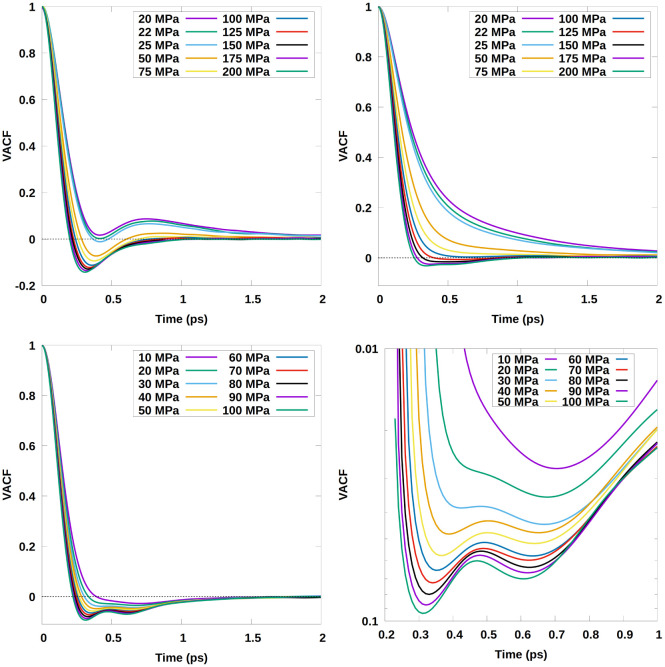
Velocity autocorrelation function from the current MLIP MD simulations. (Top Left) Atomic VACFs on the 160 K isotherm. (Top Right) Molecular VACFs on the 160 K isotherm. (Bottom Left) Molecular VACF at 90 K up to 2 ps. (Bottom Right) Molecular VACF at 90 K: zoomed-in, inverted log-scale plot of the absolute value of VACF in the relevant section, showing the appearance of a well-localized minimum, indicative of the Frenkel line crossover.

A key measurement is the velocity autocorrelation function (VACF) (Figure 6). In N_2_, there are two different relevant cases: the atomic VACF, which includes rotational correlations, and the molecular center-of-mass VACF, which excludes these.

The atomic VACF at 160 K shows a distinct minimum (Figure 6, top left panel), the position of which moves to longer times with increasing pressure up to 25 MPa and then to shorter times beyond 50 MPa. Similar behavior is seen at 90 K, with the transition at lower pressure. The crossover coincides with the onset of negative VACF. The low-pressure regime is consistent with hindered rotors, with the secondary maximum in the VACF appearing when many of the rotors have gone through 2π, or a full phase of libration.

This rotational component is absent in the center of mass VACF. There are still oscillations for pressures beyond the Frenkel line, showing that the liquid supports oscillatory motion and thereby high-frequency shear waves. The zoomed-in region in the bottom right-hand panel (Figure 6) shows the same kind of behavior for N_2_ in the current case as previously shown by Yang et al.^33^ for CO_2_, H_2_O, and CH_4_ when crossing the Frenkel line. The pressures for the crossing of the Frenkel line, indicated by the appearance of the minima (between 20 and 30 MPa at 90 K, and around 75 MPa for 160 K), are in excellent agreement with the determination using the coordination number as a marker (22.5 MPa at 90 K, 75 MPa at 160 K).

Such oscillating caged molecules contribute an additional degree of freedom to the heat capacity and a coupling of vibrational modes in adjacent molecules, leading to measurable changes in spectroscopy.

Consequently, “caged″/“rigid″ vs “uncaged″/“non-rigid″ fluid seems like a more appropriate description.

The transition in coordination number observed when crossing the Frenkel line deep in the subcritical region is harder to determine than that in the supercritical region. This can be explained by the limited range of accessible densities along an isotherm. At the triple point, the liquid density is 867 kg/m^3^, while the solid density 1026 kg/m^3^. By contrast, at the critical point the liquid density is just 313 kg/m^3^, despite the 2 orders of magnitude higher pressure (5.5 vs 0.07 MPa). Consequently, deep in the subcritical region, there is a more limited range of densities between the boiling and freezing lines.

The difficulty in collecting neutron-scattering data is ameliorated by the use of molecular dynamics simulations, whose validity is evidenced by their very close agreement with the EPSR structures. The simulations allowed us to interpolate between our experimental (*P*,*T*) data points collected over three separate campaigns.

We have also found that the coordination number is almost entirely dependent on density and is extremely well determined by a simple equation involving density at the melt line and molecular size.

We have introduced a new criterion for the Frenkel line based on the experimental angular distribution function extracted from EPSR and its simulational equivalent from molecular dynamics. This uses angular descriptors for the environment of each molecule, which map onto BCC, HCP, and FCC in the solid phase. This should not be interpreted too literally as “HCP-like″ vs “FCC-like″ fluids, but rather as an abstract measure of local configuration that shows a noticeable change along the “Frenkel″ line. We further note that, since the Frenkel “line″ is really a crossover, definitions do not need to match precisely. Equation 1 (coordination number), the HCP/FCC ratio, the triple point isotherm, the maximum in –*∂c*_*V*_/*∂T*, changes in Raman signal, and viscosity may all give slightly different “Frenkel″ lines. However, all these lines extend into the subcritical liquid and lie on the high-pressure side of the Widom lines, which are defined by extrema in second derivatives of the free energy. One can think of the Frenkel “lines″ as separating caged and uncaged liquids, in a similar way to how Widom lines separate gas-like and liquid-like fluids.
